# Widespread positive selection in the photosynthetic Rubisco enzyme

**DOI:** 10.1186/1471-2148-7-73

**Published:** 2007-05-11

**Authors:** Maxim V Kapralov, Dmitry A Filatov

**Affiliations:** 1 School of Biosciences, University of Birmingham, Edgbaston, Birmingham, B15 2TT, UK

## Abstract

**Background:**

Rubisco enzyme catalyzes the first step in net photosynthetic CO_2 _assimilation and photorespiratory carbon oxidation and is responsible for almost all carbon fixation on Earth. The large subunit of Rubisco is encoded by the chloroplast *rbcL *gene, which is widely used for reconstruction of plant phylogenies due to its conservative nature. Plant systematicists have mainly used *rbcL *paying little attention to its function, and the question whether it evolves under Darwinian selection has received little attention. The purpose of our study was to evaluate how common is positive selection in Rubisco among the phototrophs and where in the Rubisco structure does positive selection occur.

**Results:**

We searched for positive selection in *rbcL *sequences from over 3000 species representing all lineages of green plants and some lineages of other phototrophs, such as brown and red algae, diatoms, euglenids and cyanobacteria. Our molecular phylogenetic analysis found the presence of positive selection in *rbcL *of most analyzed land plants, but not in algae and cyanobacteria. The mapping of the positively selected residues on the Rubisco tertiary structure revealed that they are located in regions important for dimer-dimer, intradimer, large subunit-small subunit and Rubisco-Rubisco activase interactions, and that some of the positively selected residues are close to the active site.

**Conclusion:**

Our results demonstrate that despite its conservative nature, Rubisco evolves under positive selection in most lineages of land plants, and after billions of years of evolution Darwinian selection still fine-tunes its performance. Widespread positive selection in *rbcL *has to be taken into account when this gene is used for phylogenetic reconstructions.

## Background

Ribulose-1,5-bisphospate carboxylase/oxigenase (Rubisco; EC 4.1.1.39) is the cornerstone of photosynthesis responsible for the conversion of inorganic carbon into organic compounds. "The most abundant protein in the world", Rubisco, comprises up to 50% of all soluble proteins in plants [1], which is the price phototrophs have to pay for the enzyme's relatively slow and inefficient performance. Rubisco confuses the substrate of photosynthesis, CO_2_, with the product, O_2_, resulting in energy-wasting photorespiration. As the performance of this enzyme may greatly affect crop yields, significant efforts have been made to study the structure and the function of Rubisco, with the aim to artificially improve its performance [1]. Significant natural variation among the kinetic parameters of Rubiscos from different species [2] and its dependence mainly on environmental pressure (rather than phylogenetic factors, [3]) indicate possible positive selection to optimize CO_2_/O_2 _specificity and maximize the rate of catalytic turnover of Rubisco in different thermal and gaseous conditions [4].

Rubisco is one of the slowest and largest enzymes, with a molecular mass of 560 kDa [1]. In land plants and green algae, the chloroplast *rbcL *gene encodes the 55-kDa large subunit, whereas a family of *rbcS *nuclear genes encodes nearly identical 15-kDa small subunits [5,6]; in nongreen algae both the *rbcL *and *rbcS *genes are chloroplast encoded [7]. The Form I Rubisco of plants and algae is a hexadecameric protein composed of eight large and eight small subunits, while the Form II Rubisco of some prokaryotes and dinoflagellates consists of a large subunit dimer [7]. Because large subunits of Form II enzymes contain all the structural elements required for catalysis, the origin and the role of small subunits in Form I enzymes remain enigmatic [1].

Being one of the most conservative genes, *rbcL *is often chosen by botanists for phylogenetic reconstructions and it has been sequenced in thousands of plant species [8-10]. Surprisingly, despite Rubisco's great physiological importance, well studied protein structure and abundance of sequence data "the systematists have generally treated *rbcL *sequences as strings of anonymous nucleotides, without function" [8]. While degeneration or loss of *rbcL *in parasitic non-photosynthetic flowering plants has attracted much attention [11,12], little is known about *rbcL *evolution in other groups. The highly conservative nature of *rbcL *is probably the reason for the lack of enthusiasm for the evolutionary analysis of this gene, and in particular for the study of putative positive selection acting on *rbcL*. The only exception to this trend was the analysis of the *rbcL *evolution in the thermotolerant cyanobacteria genus *Synechococcus*, which has shown an increase in the number of hydrophobic residues in the *rbcL*s of more thermotolerant strains – a pattern observed for many thermostable proteins [13]. However, the dataset of eight sequences used in *Synechococcus *study could be too small to detect positive selection using likelihood molecular phylogenetic analysis [14].

Previously we have reported positive selection in the *rbcL *gene associated with an adaptive radiation in the Hawaiian endemic genus *Schiedea *of the *Caryophyllaceae *family [15]. Interestingly, the *Schiedea *study demonstrated that adaptive substitutions in *rbcL *could have caused the spread of an advantageous haplotype across several closely related species, causing incongruence between the chloroplast and species phylogenies [15]. However, positive selection in *rbcL *of *Schiedea *could have been driven by adaptation to contrasting environments (e.g. rainforest vs. dry coastal cliffs) and it remains unclear how common is positive selection in *rbcL *of other phototrophs. This motivated us to conduct a wider study of positive selection in the *rbcL *gene. Here we report the phylogeny-based and protein structure-based analyses of positive selection in 3228 *rbcL *sequences representing all the main lineages of green plants and some of other phototrophs, such as brown and red algae, diatoms and euglenids, as well as cyanobacteria. We found that while there is no clear evidence for positive selection in cyanobacteria and algae, positive Darwinian selection in *rbcL *is fairly common in all the main lineages of land plants (mosses, ferns and allies, gymnosperms, angiosperms). Using the computational analyses of the tertiary structure of Rubisco we show that positively selected residues are mainly located in regions important for dimer-dimer, intradimer, large subunit-small subunit and Rubisco-Rubisco activase interactions.

## Results and discussion

### Positive selection in Rubiscos of land plants, but not of algae and cyanobacteria

In order to test for the presence of positive selection acting on Rubisco we used 3228 *rbcL *sequences from different phototrophs (Additional file 1). Most *rbcL *sequences analyzed (80%) belong to flowering plants and represent 43 orders and 203 families (96% of flowering plant orders and 44% of families *sensu *APG II [10]) providing reasonable coverage of the most taxon-rich lineage of phototrophs. The coverage outside flowering plants was less extensive (Table 1 and Additional file 1). For computational efficiency all the sequences were divided into 151 monophyletic groups, based on their phylogenetic relations (see methods and Additional file 1).

**Table 1:** Sampled groups

Group	N orders	N families	N genera	N sequences
**The Plantae Kingdom (plants)**				
Magnoliophyta (angiosperms)	43	203	1544	2572
Pinophyta and Gnetophyta (gymnosperms)	3	6	40	201
Filicophyta (ferns)	1	10	39	156
Lycopodiophyta (clubmosses)	1	1	3	27
Equisetophyta (horsetails)	1	1	1	19
Bryophyta (mosses)	8	20	42	88
Charophyta (green algae)	1	1	6	49
**Plastid-carrying eukaryotes which are not in the Plantae Kingdom**				
Phaeophyta (brown algae)	4	11	30	48
Bacillariophyta (diatoms)	6	10	15	20
Rhodophyta (red algae)	6	10	14	20
Euglenida (euglenid protists)	2	2	5	11
**Prokaryotes**				
Cyanobacteria	2	4	8	17
**Sum**	78	279	1747	3228

For the detection of positive selection we used nested maximum likelihood models allowing for variation in the ratio of non-synonymous to synonymous substitutions rates (d*N*/d*S*) across codons implemented in PAML [16,17]. For each dataset we performed one Likelihood Ratio Test (LRT) for d*N*/d*S *heterogeneity across codons (M0-M3 comparison, [17]), which was significant for all 151 analyzed groups, indicating expected heterogeneity in selective pressure across the *rbcL *molecule (data not shown). Further, we performed two LRTs for the presence of codons under positive selection: M7-M8 [17] and M8a-M8 comparisons [18]. The M7 model assumes a discrete beta distribution for d*N*/d*S*, which is constrained between 0 and 1, implemented using ten classes taken in equal proportions. To test for the presence of codons with d*N*/d*S *> 1, M7 is compared to the M8 model, which is similar to the M7 model, but allows for an extra "eleventh" class with d*N*/d*S *≥ 1 [17]. This test was significant for 119 out of 151 analyzed groups (Table 2 and Additional file 2). A more stringent test for positive selection compares model M8 with M8a, which is similar to the model M7, but allows for an extra class of codons with d*N*/d*S *= 1 [18]. This test was significant for 121 out of 151 analyzed groups (Tables 2 and Additional file 2). In 112 cases (74%) both M7-M8 and M8a-M8 comparisons rejected models without positive selection in favor of M8 model assuming positive selection (Table 2 and Additional file 2).

**Table 2:** LRT summary statistics

						11th class from M8		M7-M8		M8a-M8		Both LRTs	
						
Lineage	N ^c^	Tree length ^d^	dS ^e^	dN ^f^	M0 dN/dS ^g^	p,%	dN/dS	Np ^i^	% ^j^	Np ^I^	% ^j^	Np ^I^	% ^j^
angiosperms	122	0.56 (0.31)	0.55 (0.34)	0.07 (0.04)	0.17 (0.12)	3.4 (2.4)	5.23 (6.56)	96	79	103	84	95	78
gymnosperms	8	0.27 (0.27)	0.26 (0.31)	0.04 (0.02)	0.23 (0.12)	3.2 (2.7)	8.57 (7.13)	7	88	8	100	7	88
ferns and allies ^a^	9	0.96 (1.05)	0.95 (1.06)	0.07 (0.05)	0.10 (0.05)	1.4 (1.0)	4.15 (1.68)	9	100	7	78	7	78
mosses	4	0.75 (0.48)	1.35 (0.87)	0.06 (0.04)	0.04 (0.00)	1.6 (2.1)	3.77 (2.56)	4	100	3	75	3	75
algae ^b^	7	3.49 (2.94)	6.33 (6.25)	0.15 (0.12)	0.03 (0.03)	0.0	n.a.	2	29	0	0	0	0
cyanobacteria	1	2.65	3.01	0.12	0.04	1.9	1.02	1	100	0	0	0	0
all	151	0.72 (0.95)	0.87 (1.81)	0.07 (0.05)	0.16 (0.12)	3.1 (2.4)	5.21 (6.31)	119	79	121	80	112	74

In the columns three to eight mean values and standard deviations (in brackets) are given.

^a ^Including Filicophyta, Lycopodiophyta and Equisetophyta.

^b ^Including Charophyta, Phaeophyta, Bacillariophyta, Rhodophyta and Euglenida.

^c ^Number of groups analyzed.

^d ^Estimated using M0 model in PAML.

^e ^The rate of synonymous substitutions per synonymous site calculated using M0 model in PAML.

^f ^The rate of nonsynonymous substitutions per nonsynonymous site calculated using M0 model in PAML.

^g ^The ratio of non-synonymous to synonymous substitutions rates (dN/dS) calculated using M0 model in PAML.

^i ^Number of groups with detected positive selection in *rbcL *among investigated plant lineages. *P *< 0.05

^j ^Proportion of groups with detected positive selection in *rbcL *among investigated plant lineages. *P *< 0.05

For all analyzed lineages of land plants (mosses, ferns and allies, gymnosperms, angiosperms) positive selection was found in most cases (Table 2). The smallest proportion of cases with detected positive selection and average dN/dS value of "eleventh" class in M8 model were in mosses (75% and 3.8 respectively); the highest – in gymnosperms (88% and 8.6 respectively). There was no significant difference between the lineages of land plants in proportion of groups with positive selection (2 × 2 contingency χ^2 ^tests with Yates' correction). Among the main lineages of flowering plants – magnoliids, monocots, commelinids, eudicots (including eudicots and core eudicots), rosids (including rosids, rosids I and rosids II) and asterids (including asterids, euasterids I and euasterids II; all groups *sensu *APG II [10]) – the smallest proportion of cases with detected positive selection was in monocots (61%); the highest – in asterids (89%) (Additional file 2). There was no significant difference between the lineages of flowering plants in proportion of groups with positive selection (2 × 2 contingency χ^2 ^tests with Yates' correction).

While positive selection is widespread in land plants we did not find it in algae (including green, brown and red algae, diatoms and euglenids) and cyanobacteria (Table 2). Positive selection has been detected in a combined *rbcL *dataset of 500 sequences of land plants and algae (M. Anisimova and V. Savolainen, personal communication). The fact that positive selection was detected in a large combined data set may simply mean that the signal of positive selection is very strong in land plants so M. Anisimova and V. Savolainen still detect the signal when averaged across lineages with no positive selection, such as algae.

As increasing number of sequences should increase the sensitivity of the analysis [14], we joined the sets of green (Charophyta-1 + Charophyta-2, 49 sequences in total) and brown algae (Phaeophyta-1 + Phaeophyta-2, 48 sequences in total). Again there was no evidence for positive selection in the joint algae dataset in either M7-M8 or M8a-M8 comparisons. Although in our dataset land plants were much better represented (143 groups), compared to algae (7 groups) and cyanobacteria (1 group), the difference in proportion of groups with positive selection was significantly larger in land plants than in algae and cyanobacteria combined (2 × 2 contingency χ^2 ^with Yates' correction = 20.3, *P *< 0.00001). In fact, the difference in proportion of groups with positive selection between land plants and algae is conservative, given the sequence divergence is higher in algae and cyanobacteria datasets, compared to land plants (Table 2). The power of LRTs increases with sequence divergence until it reaches its maximal value, after which further increases of sequence divergence lead to reduced power [14]. The tree length values (the expected number of nucleotide substitutions per codon along the tree) for algae and cyanobacteria datasets ranged from 1.3 to 8.6 and were in the optimal range for detection of positive selection [14]. The tree length values for the most of the land plant datasets were smaller than optimal (< 1; Table 2 and Additional file 2), hence the number of cases with detected positive selection in land plants may be underestimated.

Could the difference in the presence of positive selection between land plants and algae be due to structural differences of their Rubiscos? There are prominent differences in the Rubisco protein structures within the paraphyletic algae group with no positive selection detected (the "green-like" Rubiscos of green algae, euglenids and cyanobacteria vs. the "red-like" Rubiscos of red and brown algae and diatoms), while the Rubisco structures of land plants with widespread positive selection and green algae with no selection detected are virtually identical [7]. So, structural differences can not explain the contrast between algae that do not show evidence for positive selection in *rbcL*, and land plants, where positive selection in this gene is ubiquitous.

An aquatic habitat is one of the few parameters shared by most algae and cyanobacteria, but not by most of land plants, thus the presence of positive selection in Rubiscos of land plants, but not in algae and cyanobacteria may be due to ecological differences between aquatic and terrestral habitats. Thermal and water regimes are more stable in aquatic, compared to terrestrial environments. Furthermore, algae have a "bicarbonate pump" – the CO_2_-concentrating mechanism that uses bicarbonate dissolved in water and suppresses the oxygenating activity of Rubisco, making gaseous conditions of Rubisco performance more stable [19]. Interestingly, aquatic land plants, a small group of angiosperms with a submerged aquatic lifestyle as a derived state, also use the bicarbonate pump [19] and hence may be expected to have weaker signal of positive selection than their terrestrial relatives. Indeed the sets that consisted exclusively of submerged aquatic angiosperms (monocots-4 and 9) or contained a high proportion of them (monocots-5 and 7) did not show any evidence for positive selection in *rbcL *(Additional file 2). However, when aquatic land plants from sets monocots-4 and 9 were analyzed together (including submerged aquatic plants from other monocot groups) both M7-M8 and M8a-M8 comparisons confirm a presence of positive selection, suggesting that its signal in aquatic land plants is too weak to be detected in the analyses of smaller groups (joint group of submerged aquatic monocot plants: N sequences = 67; M7-M8: χ^2 ^= 26.72, *P *= 0.00001; M8a-M8: χ^2 ^= 4.78, *P *= 0.0144). Thus, it seems likely that aquatic conditions require less fine-tuning of Rubisco activity by positive selection, compared to terrestrial habitats.

Our analysis of 3228 *rbcL *sequences revealed the presence of positive selection in 78% of analyzed land plant groups, but not in algae and cyanobacteria. The finding of widespread positive selection in Rubisco suggests that either selection still continues to improve performance of this ancient critically important enzyme, or that adaptive evolution in *rbcL *may reflect the fine-tuning of Rubisco to optimize its performance in various gaseous and thermal conditions [4]. The much weaker evidence for positive selection in algae and aquatic land plants growing in more stable conditions, compared to terrestrial land plants, suggests that the later explanation is more probable. This is also consistent with the finding that natural variation among the kinetic parameters of Rubisco enzymes from different species depends mainly on environmental pressures rather than on the phylogeny [3].

### A few Rubisco residues are responsible for the most cases of positive selection

**Figure 1:**
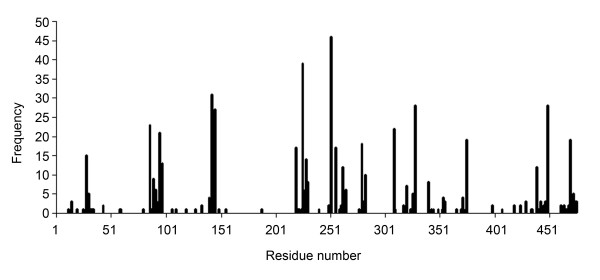
**The distribution of *rbcL *residues evolving under positive selection**. The distribution of residues identified in our analyses as evolving under positive selection in 112 groups with positive selection in *rbcL*. Shown are the residues with Bayesian posterior probability of positive selection larger than 0.95, when analyzed by the Bayes Empirical Bayes [20]. Numbering of residues is after the spinach sequence.

**Figure 2:**
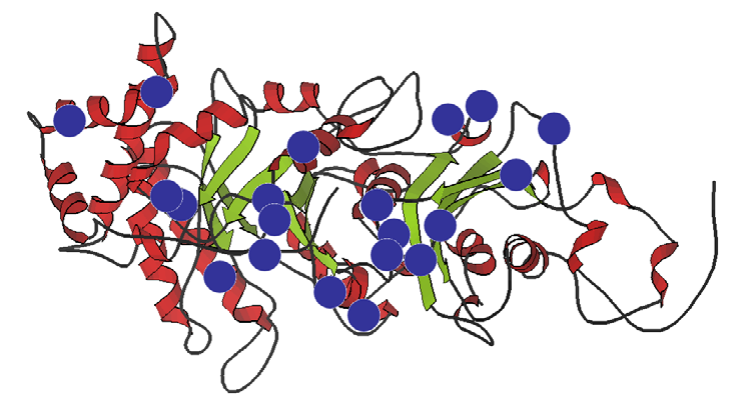
**Locations of the twenty most often positively selected Rubisco residues**. The large subunit of spinach Rubisco is shown (chain L) after [37] with locations of the twenty most often positively selected Rubisco residues (Table 3) highlighted by blue circles. Visualization is made using the KiNG viewer [41].

To identify amino acid sites potentially under selection in the groups with positive selection, the parameter estimates from M8 model were used to calculate the posterior probabilities that a codon belongs to a class with d*N*/d*S *> 1 using the Bayes Empirical Bayes approaches implemented in PAML [20]. In 112 groups with positive selection detected by both M7-M8 and M8a-M8 comparisons, 98 out of 476 Rubisco residues (Additional file 3) had a Bayesian posterior probability of positive selection larger than 0.95 in one or more cases when analyzed by the Bayes Empirical Bayes [20]. In 106 groups (95%) more than one residue was under selection (average number of amino acids under selection per group was 5.4 ± 3.3). The distribution of residues identified in our analyses as evolving under positive selection was highly uneven: twenty of the most often selected residues are responsible for more than 70% of the cases of positive selection (Figure 1, Table 3 and Additional file 3). Analyses of Rubisco tertiary structure revealed that some of the twenty most often selected residues are quite close to each other and most of them are involved in interactions between Rubisco large and small subunits, in interactions with Rubisco activase, dimer-dimer and intradimer interactions, as well as in interactions with the active site (Figure 2, Table 3). The analyses of mutant Rubisco enzymes have shown that interface between large and small subunits contributes to holoenzyme thermal stability, catalytic efficiency, and CO_2_/O_2 _specificity [21,22]. Rubisco activase is responsible for facilitating the opening of the closed Rubisco form to release ribulose-1,5-bisphospate and to produce the active form of the enzyme [1,23,24]. Loop 6 plays a major role in discriminating between CO_2 _and O_2 _and functions as a flexible "flap" that closes over the active site once the substrates are bound, and the carboxyl terminus folds over loop 6 and appears to stabilize its closed conformation [25]. More specifically, the effects of amino acid replacements in three residues (number 86, 262, 449) out of twenty most selected in our analyses were tested by directed mutagenesis in the green alga *Chlamydomonas reinhardtii*: aspartate 86 to arginine substitution had little effect [23]; valine 262 to leucine substitution improved the termal stability of wild-type Rubisco in vitro [21]; cystein 449 to serine substitution showed an increased resistance to inactivation when Rubisco in the oxidized state [26]. The general congruence between our findings and ones obtained by mutagenic approach suggests that amino acids evolving under positive selection in *rbcL *are located in regions important for Rubisco activity and residues involved in dimer-dimer, intradimer, large subunit-small subunit and Rubisco-Rubisco activase interactions as well as ones close to the active site are apparently the prime targets of positive selection in Rubisco. The Rubisco regions characterized by high density of residues evolving under positive selection and located relatively far away from the active site (e.g. strands C-D region, helix D, helix 2, helix 3; table 3) could be good candidates for mutagenic studies to reveal the broader picture of how Rubisco functions. Detection of positive selection at the interfaces between chloroplast- and nuclear-encoded Rubisco subunits and between Rubisco and Rubisco activase suggests that co-evolution of proteins in the Rubisco complex can be another driving force of adaptive evolution in *rbcL*.

**Table 3:** Twenty most often positively selected rbcL residues

Residue No ^1^	N ^2^	Location of residue	Residues within 5 Å ^3^	Structural motifs within 5 Å	Interactions^4^
251	46	helix 3	247, 248, 249, 250, 252, 253, 254, **255**, 256, **279**, 280, 283	helixes 3, 4	DD, *SSU*
225	39	helix 2	189, 190, 193, 194, 221, 222, 223, 224, 226, 227, **228**, 229, 236, 237, 238	helixes 1, 2; strand 3	SSU
142	31	helix D	33, 140, 141, 143, 144, **145**, 146, 367, 369	N-terminus; strands D, H	DD
328	28	loop 6	295, 311, 326, 327, 329, 330, 342, 345, 346, 349, 376, 377, 378, 394	AS; loop 6 region; helixes 5, 7; strand 7	AS
449	28	helix G	445, 446, 447, 448, 450, 451, 452, 453, 455, 456	C-terminus	SSU
145	27	helix D	140, 141, **142**, 143, 144, 146, 147, 148, 320, 366, 367, 368, 369, 371	helixes D, 5, H	DD
86	23	strand C	25, 27, 84, 85, 87, 88, 98, 99, 100	strands A, C, D	*RA*
309	22	strand F	117, 121, 125, 134, 135, 301, 302, 307, 308, 310, 311, 313, 314	strand E; helixes F, 5	ID
95	21		42, 43, 44, 93, 94, 96, **97**, 131	strands B, D, E	ID, *RA*
375	19	strand 7	155, 158, 159, 169, 324, 325, 326, 373, 374, 376, 377, 397, 398, 399	helix E; strands 6, 7, 8	SSU
470	19	C-terminus	336, 468, 469, 471, 472	loop 6; C-terminus	ID, *RA*
279	18	helix 4	250, **251**, 274, 275, 276, 277, 278, 280, 281, **282**, 283	helixes 3, 4	
219	17	helix 2	58_I_, 59_I_, 61_I_, 214, 215, 216, 217, 218, 219, 220, 221, 222, 223, 256, 260	helixes 2, 3	SSU, DD
255	17	helix 3	**251**, 252, 253, 254, 256, 257, 258, 259, 283	helixes 3, 4	SSU, DD
28	15	N-terminus	25, 26, 27, 29, 30, 84	strands A, C	
228	14	helix 2	190, 193, 194, 224, **225**, 226, 227, 229, 230, 231, 232, 236	helixes 1, 2	SSU
97	13	strand D	40, 41, 42, 44, 50, 87, 88, 89, 90, **95**, 96, 98, 99, 100	helix B; strands B, C, D	*RA*
262	12		59_S_, 60_S_, 226, 240, 257, 258, 260, 261, 263, 264, 289	helixes 2, 3; strand 3	SSU, DD
439	12	helix G	436, 437, 438, 440, 441	helix G	
282	10	helix 4	149, 278, **279**, 280, 281, 283, 284, 285, 286, 321	helixes 4, 5	DD, *SSU*

^1 ^Numbering of residues is after the spinach Rubisco sequence.

^2 ^Number of groups with detected signal of positive selection where the particular residue was shown under positive selection with Bayesian posterior probability larger than 0.95, when analyzed by the Bayes Empirical Bayes [20].

^3 ^Subscriptions denote residues from I and S small subunits. Residues within the list of the twenty designated residues are underlined.

^4 ^Interactions in which the twenty selected residues and/or residues within 5 Å of them are involved. AS – interactions with the active site; ID – intradimer interactions; DD – dimer-dimer interactions; RA – interface for interactions with Rubisco activase; SSU – interactions with small subunits; after [8]. Interactions based on literature survey only are given in italics; after [1,23,27].

Directed mutagenesis of Rubisco residues in *Chlamydomonas *showed that a single amino acid substitution, apart from the active site, has little effect on Rubisco function even if it occurs in a functionally important region, but when double or triple substitutions were created, significant changes were observed in both enzyme catalytic efficiency and specificity [1,22,26,27]. In our study, in 95% of groups with detected positive selection, several residues (> 5 on average) simultaneously were shown to evolve under positive selection (Additional file 3) suggesting their coevolution within Rubisco. Simultaneous coevolution of multiple residues makes mutagenic studies of Rubisco functioning more laborious as with any new residue added the number of mutant combinations to be tested rises exponentially. However the integration of phylogenetic and biochemical approaches might be beneficial for understanding of Rubisco functional mechanisms. As natural variation among the kinetic parameters of Rubiscos from different species mainly depends on environmental pressure rather than phylogenetic factors [3], the integration of Rubisco activity essays with molecular phylogenetic analysis in a group of closely related plant species occupying contrasting environments could give direct evidence of how Rubisco evolves in nature.

### Implications for phylogenetic studies

Although *rbcL *was used in hundreds of phylogenetic studies, only in a small fraction of them was it treated as a biological molecule, not as "strings of anonymous nucleotides, without function" [8]. Our analysis demonstrated that *rbcL *can not be regarded as a neutral marker and positive selection is fairly common in this gene. Källersjö and coauthors [9] simultaneously analyzed 2538 *rbcL *sequences by parsimony jackknifing and found that the first and second codon positions together contain 764 informative positions which support 431 clades, whereas 471 informative third positions support 1327 clades, clearly showing that the third positions on average have a stronger phylogenetic signal. These findings do not support frequent assumption that when changes occur more often in the third position, they are likely to contain more homoplasy and provide less phylogenetically reliable information then more conservative first and second positions [9]. Positive selection may also result in homoplasy due to fixations of the same mutation that arose independently in several phylogenetic lineages. As most substitutions in the third codon positions are synonymous, the third codon positions are less frequent targets of positive selection compared to the first and second ones. Thus, findings that the first and second codon positions in *rbcL *have a lesser phylogenetic signal then expected [9,28] can be explained by widespread positive selection on *rbcL*.

We tested whether the removal of codons evolving under positive selection will improve phylogenetic resolution in 112 groups with detected positive selection (Additional file 4). We compared sums of bootstrap values between the trees reconstructed using all sites and the trees reconstructed using only neutrally evolving sites (positively selected sites were excluded). The sums of bootstrap frequencies did not increase or decrease for more than 5% in 41% of analyzed cases; decreased for more than 5% in 29% of cases, and increased for more than 5% in 30% of cases. In two cases, deletion of codons evolving under positive selection resulted in more than 55% increase of the total bootstrap support (Additional file 4). Thus, taking into account the presence of positive selection in *rbcL *may improve phylogenetic reconstructions. We recommend checking *rbcL *datasets for positive selection, and if selection is found, to test whether deletion of sites evolving under positive selection from further phylogenetic analyses would increase topological resolution/bootstrap support of the selected branches. Alternatively, sites evolving under positive selection could be appropriately modelled for improving their contribution into phylogenetic signal, although there is no available "ready to use" program which can do it at the moment.

Adaptive mutations may spread across subpopulations of a species, or across several species with very little gene flow [29]. Thus, positive selection in Rubisco may facilitate horizontal interspecific gene flow for chloroplast DNA, as spreading of adaptive mutations in *rbcL *may result in fixation of a single chloroplast haplotype in several occasionally hybridising species, which may dramatically affect phylogeny reconstruction. Previously we detected strong cytonuclear discordance apparently caused by positive selection in *rbcL *in the Hawaiian endemic plant genus *Schiedea *[15]. This illustrates the danger of reconstructing phylogenetic relations solely from chloroplast data in groups with putative interspecific hybridization: tests for the presence of positive selection and for the congruence between chloroplast and nuclear phylogenies are essential for correct inference of species phylogenetic relations.

## Conclusion

Our molecular phylogenetic analysis unexpectedly revealed that positive selection in the *rbcL *gene of terrestrial land plants is quite a common phenomenon. On the other hand, positive selection in cyanobacteria, algae and aquatic land plants is less prominent, which may possibly be explained by more stable conditions of aquatic environment compared to terrestrial one. The residues involved in dimer-dimer, intradimer, large subunit-small subunit and Rubisco-Rubisco activase interactions as well as ones close to the active site are apparently the prime targets of positive selection in Rubisco. Widespread adaptive evolution in *rbcL *may reflect the perpetual fine-tuning of Rubisco to optimize its performance in changing gaseous and thermal conditions and/or co-evolution of proteins in the Rubisco complex. The integration of phylogenetic and biochemical research is required to test the hypothesis that Darwinian selection during Rubisco evolution is driven by continuous fine-tuning to changing conditions. Widespread positive selection in *rbcL *has to be taken into account when this gene is used for phylogenetic reconstructions specifically when interspecific hybridization is possible.

## Methods

### Data preparation

All but thirteen *rbcL *sequences used in this study were extracted from NCBI GenBank [30] and the species names and accession numbers as well as taxonomic information are given in Additional file 1. The thirteen novel sequences (all representing genus *Silene*) were deposited in [GenBank:EF418555–EF418567].

The obtained sequences were aligned and edited for further analyses using ProSeq3 software [31]. Codon alignments were made from amino-acid alignments and manually checked. All alignments were straightforward and unambiguous confirming the highly conservative nature of *rbcL*. We found only one insertion/deletion which separated the group of red, brown and diatom algae from all the rest of analyzed lineages. This indel gap did not impact our analyses because we analyzed many separate data sets instead of the joint one (see below). It should be noted that many sequences lack bases at the 5' and/or the 3' end; we made alignments within each analyzed dataset of the same length by sequence truncation. Suspicious sequences (such as containing stop codons) were not included into analyses. All alignments are available upon request from the corresponding author.

### Likelihood ratio tests for positive selection

For detection of positive selection we used codon-based analysis (codeml) implemented in PAML v.3.14 package [16]. It has been shown that the power to detect positive selection is close to 100% in data sets of ≥ 17 sequences [14]. As the computing time grows dramatically with the number of sequences analyzed, we divided all *rbcL *sequences into 151 relatively small monophyletic groups (Additional file 1) by manual dissection of phylogenetic trees constructed using neighbor-joining algorithm implemented in MEGA v3.1 [32].

For all analyses of positive selection we used the codeml program from the PAML package [16]. All PAML analyses were performed using "user tree" runmode in codeml. The employed trees were reconstructed by neighbor-joining algorithm implemented in MEGA [32] using following parameters: pairwise deletion of missed sites, all three codon positions used, both transitions and transversions used, homogeneous pattern among lineages and uniform rates among sites, both Kimura's [33] and Tamura-Nei's [34] models of nucleotide substitutions were used, which resulted in similar topologies. The resulting topologies were manually checked for congruence with systematics of analyzed taxa. Although some of these phylogenies may slightly deviate from the "true" species trees, this should not significantly influence Likelihood Ratio Tests (LRTs) we used for analysis of positive selection, as they were shown to be robust to phylogenetic uncertainty [35]. We used models of codon evolution that allow for variation in d*N*/d*S *among codons [17] to perform LRTs for rate heterogeneity among amino acid sites and for positive selection. We performed one LRT for d*N*/d*S *heterogeneity (M0-M3 [17]) and two LRTs for positive selection: M7-M8 [17] and M8a-M8 [18]. For all LRTs, the first model is a simplified version of the second one, with fewer parameters, and is thus expected to provide a poorer fit to the data (lower maximum likelihood). The M7 and M8a models are the null models without positive selection (no codons with d*N*/d*S *> 1) and the M8 model is the alternative model with positive selection. The significance of the LRTs was calculated assuming that twice the difference in the log of maximum likelihood values between the two models is distributed as a χ^2 ^distribution. The degrees of freedom (df) were given by the difference in the numbers of parameters in the two nested models. It was argued that for the M0-M3 and M7-M8 comparisons the df = 2 [17], while for M8a-M8 comparisons the appropriate test would use a 50:50 mixture of df = 0 and df = 1 [18]. Accordingly, to calculate a *P*-value from this mixture of distributions we first calculated the *P*-value assuming df = 1, and then halved it. Cases in which M8 model fitted better with *P *< 0.05 in both M7-M8 and M8a-M8 comparisons were regarded as having positive selection.

The significance of difference in proportion of cases with detected positive selection between the analyzed plant groups was evaluated by 2 × 2 contingency χ^2 ^tests with Yates' correction. First, we performed pairwise comparisons between four groups of land plants (mosses, ferns and allies, gymnosperms, angiosperms); second, between six groups of angiosperms (magnoliids, monocots, commelinids, eudicots, rosids, asterids; all groups *sensu *APG II [10]); and, finally, between land plants and combined algae and cyanobacteria dataset.

To identify amino acid sites potentially under selection in the groups with confirmed positive selection, the parameter estimates from M8 model were used to calculate the posterior probabilities that a codon belongs to a class with d*N*/d*S *> 1 using the Bayes Empirical Bayes approaches implemented in PAML [20].

### Structural analysis of Rubisco

The analyzed *rbcL *sequences are fairly conserved, and any differences in length occur at the C-terminus. This allows us to use published spinach Rubisco protein structure [36-38] for structural analysis. Throughout the paper, the numbering of Rubisco large subunit residues is based on the spinach sequence. Rubisco structural data files for spinach 1RBO [37] and 1RCX [38] were obtained from the RCSB Protein Data Bank [39]. The locations and properties of individual amino acids in the Rubisco structure were analyzed using DeepView – Swiss-PdbViewer v.3.7 [40].

### Evaluation of effects of positive selection on phylogenetic reconstructions

Given that positive selection may result in homoplasy we tested whether the removal of codons evolving under positive selection will improve the phylogenetic resolution. We compared boostrap sums of trees reconstructed using all sites (including ones evolving under positive selection) with boostrap sums of trees reconstructed using only neutrally evolving sites. Phylogenetic trees were reconstructed in MEGA [32] using neighbor-joining algorithm with Tamura-Nei's [34] model of nucleotide substitutions. We used 50% majority rule trees and subtracted 50% from each support value before summing up. The subtraction was done to circumvent the bias in summing up bootstrap values of a consensus tree; e.g. a tree with two 51% groups would have higher support than one with one group with 100% support, and if support was decreased from 51% to 49%, the sum would be zero (due to a threshold of 50%).

## Authors' contributions

MK and DF conceived the study and participated in its design. MK carried out all analyses and drafted the manuscript. DF edited the manuscript. Both authors read and approved the final manuscript.

## Supplementary Material

Additional file 1 **Sampling design**. List of 151 analyzed groups is provided including taxonomic information and GenBank accession numbers of *rbcL *sequences. Click here for file

Additional file 2 **LRT statistics for 151 analyzed groups**. Likelihood Ratio Tests statistics are provided for 151 analyzed groups. Tree length, dS (the rate of synonymous substitutions per synonymous site) and dN (the rate of nonsynonymous substitutions per nonsynonymous site) and their ratio (dN/dS) were calculated using M0 model in PAML [17]; proportion of residues with dN/dS > 1 ("eleventh" class) and their dN/dS value were calculated in M8 model [17]. Twice the difference in the log of maximum likelihood values for M7-M8 and M8a-M8 comparisons and significance of the LRTs (*P*-value) are shown. Click here for file

Additional file 3 ***RbcL *residues under positive selection**. Shown are the residues of the 112 groups with confirmed positive selection in *rbcL *with Bayesian posterior probability of positive selection larger than 0.95, when analyzed by the Bayes Empirical Bayes [20]. Click here for file

Additional file 4 **Impact of sites evolving under positive selection on phylogenetic resolution**. Bootstrap frequencies sums of 50% majority rule trees before and after removal of codons evolving under positive selection are shown for 112 groups with detected positive selection. 50% were subtracted from each support value before summing up to avoid bias. Click here for file
